# Primary Gastrointestinal Stromal Tumor of the Pancreatic Head: A Diagnostic Challenge Mimicking a Neuroendocrine Tumor

**DOI:** 10.7759/cureus.99881

**Published:** 2025-12-22

**Authors:** Alejandro Hernández-Alejo, Diego Salinas-Rodríguez, Katia Sofía Alatorre-Plascencia, Guillermo Elizondo-Riojas

**Affiliations:** 1 Radiology, Hospital Universitario "Dr. José Eleuterio González", Monterrey, MEX

**Keywords:** dog-1, gastrointestinal stromal tumor (gist), neuroendocrine tumor, pancreatic extragastrointestinal stromal tumor (egist), pancreatic head masses

## Abstract

Pancreatic hypervascular masses can be difficult to characterize on cross-sectional imaging, particularly when different entities share similar enhancement patterns. We report the case of a 63-year-old woman with a three-month history of abdominal pain and progressive distension. Contrast-enhanced computed tomography (CT) revealed a 49 × 60 mm irregular hypervascular mass in the pancreatic head with peripheral arterial-phase enhancement, a large central low-attenuation area compatible with necrosis, and mass effect with displacement of the first and second portions of the duodenum without luminal obstruction or mural invasion, associated with prominent gastroduodenal veins and early portal vein opacification. Based on the lesion’s location and enhancement pattern, the initial radiologic impression was that of a pancreatic neuroendocrine tumor (NET). At laparotomy, the procedure was aborted because of severe intraoperative hemorrhage related to tumor hypervascularity, and only an incisional biopsy was obtained. Histopathology showed a mesenchymal neoplasm with a mixed spindle-cell and epithelioid pattern arranged in intersecting fascicles without necrosis. Immunohistochemistry demonstrated strong, diffuse DOG1 expression and negativity for epithelial, muscular, endothelial, and neural markers, with a Ki-67 index of approximately 1%, consistent with a low-risk gastrointestinal stromal tumor (GIST). After multidisciplinary discussion, pancreaticoduodenectomy and tyrosine kinase inhibitor therapy were proposed, but the patient declined and has been managed conservatively with close follow-up and symptomatic improvement. In this patient, the pancreatic mass mimicked a NET on imaging and was ultimately identified as an extragastrointestinal stromal tumor (GIST), underscoring the importance of histopathologic and immunohistochemical confirmation when evaluating hypervascular pancreatic lesions.

## Introduction

Gastrointestinal stromal tumors (GISTs) are the most frequent mesenchymal neoplasms of the digestive tract [1]. They can arise in any segment of the digestive tract, although they occur most commonly in the stomach and small intestine [1]. From a histogenetic standpoint, GISTs are thought to derive from the interstitial cells of Cajal or from precursors with a similar phenotype, which connect the myenteric plexus with the muscularis propria and act as pacemaker cells of the gastrointestinal tract [2]. At the molecular level, Hirota et al. demonstrated activating mutations in the proto-oncogene c-KIT in a significant proportion of these tumors, leading to constitutive activation of the KIT receptor tyrosine kinase and accounting for their proliferative behavior and response to tyrosine kinase inhibitors [3]. Consistent with this, GISTs typically show immunohistochemical expression of CD117 (KIT) and CD34, with spindle-cell, epithelioid, or mixed morphologic patterns [1]. In addition to tumors arising from the wall of the digestive tract, neoplasms with equivalent morphology and immunophenotype have been described in the omentum, mesentery, and retroperitoneum, without demonstrable continuity with the intestinal wall; these are grouped under the term extragastrointestinal stromal tumors (EGISTs) [4].

Primary involvement of the pancreas by a stromal tumor is exceptional and has been described only in small series and isolated case reports [4,5]. In an analysis of 45 patients with pancreatic EGIST identified in the literature and at a reference center, Liu et al. showed that the pancreatic head is the most frequent location, that most tumors measure more than 5 cm at diagnosis, and that a substantial proportion fall into intermediate- or high-risk categories according to size and mitotic index [5]. Radiologically, pancreatic EGISTs and neuroendocrine tumors (NETs) may both appear as enlarging, solid, or solid-cystic hypervascular masses in the pancreatic head on computed tomography (CT); they can easily be misinterpreted as one another, making histologic confirmation indispensable to guide management and to avoid misclassification that may have important implications for both prognosis and treatment planning. [5,6]. Accordingly, distinguishing pancreatic EGISTs from NETs is clinically important because, unlike neuroendocrine neoplasms, risk-adapted management of GIST incorporates KIT-targeted tyrosine kinase inhibitors, so an incorrect classification may lead to different decisions regarding systemic therapy and follow-up [1,3,5]. In this context, immunohistochemical markers play a central role: in addition to CD117, the DOG1 antigen has emerged as a sensitive and specific marker for GIST, useful even in tumors with weak or absent KIT expression [7], and reports of pancreatic EGIST emphasize strong DOG1 positivity in neoplastic cells as a key finding to confirm the diagnosis against other solid pancreatic neoplasms, particularly NETs [5,6]. Because pancreatic EGISTs and NETs may both appear as enlarging hypervascular masses in the pancreatic head on CT, they can easily be misinterpreted as one another [6]. Against this background, the present report describes a rare primary EGIST of the pancreatic head that radiologically mimicked a pancreatic NET because of its hypervascular enhancement pattern, illustrating the diagnostic challenge of distinguishing between these entities on cross-sectional imaging.

## Case presentation

A 63-year-old woman with no known oncologic history presented with abdominal pain and progressive abdominal distension of approximately three months’ duration. She had not undergone prior imaging studies.

**Figure 1:**
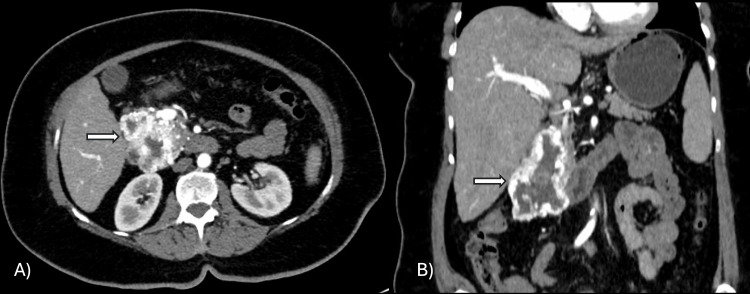
Contrast-enhanced CT scan of the abdomen in the arterial phase Axial (A) and coronal (B) sections showing a solid hypervascular mass in the pancreatic head with irregular margins (arrow), peripheral enhancement, and a large central low-attenuation area compatible with necrosis. CT: computed tomography

At initial evaluation, contrast-enhanced CT of the abdomen with intravenous contrast showed an irregular mass in the pancreatic head measuring approximately 49 × 60 mm (Figure 1).

**Figure 2:**
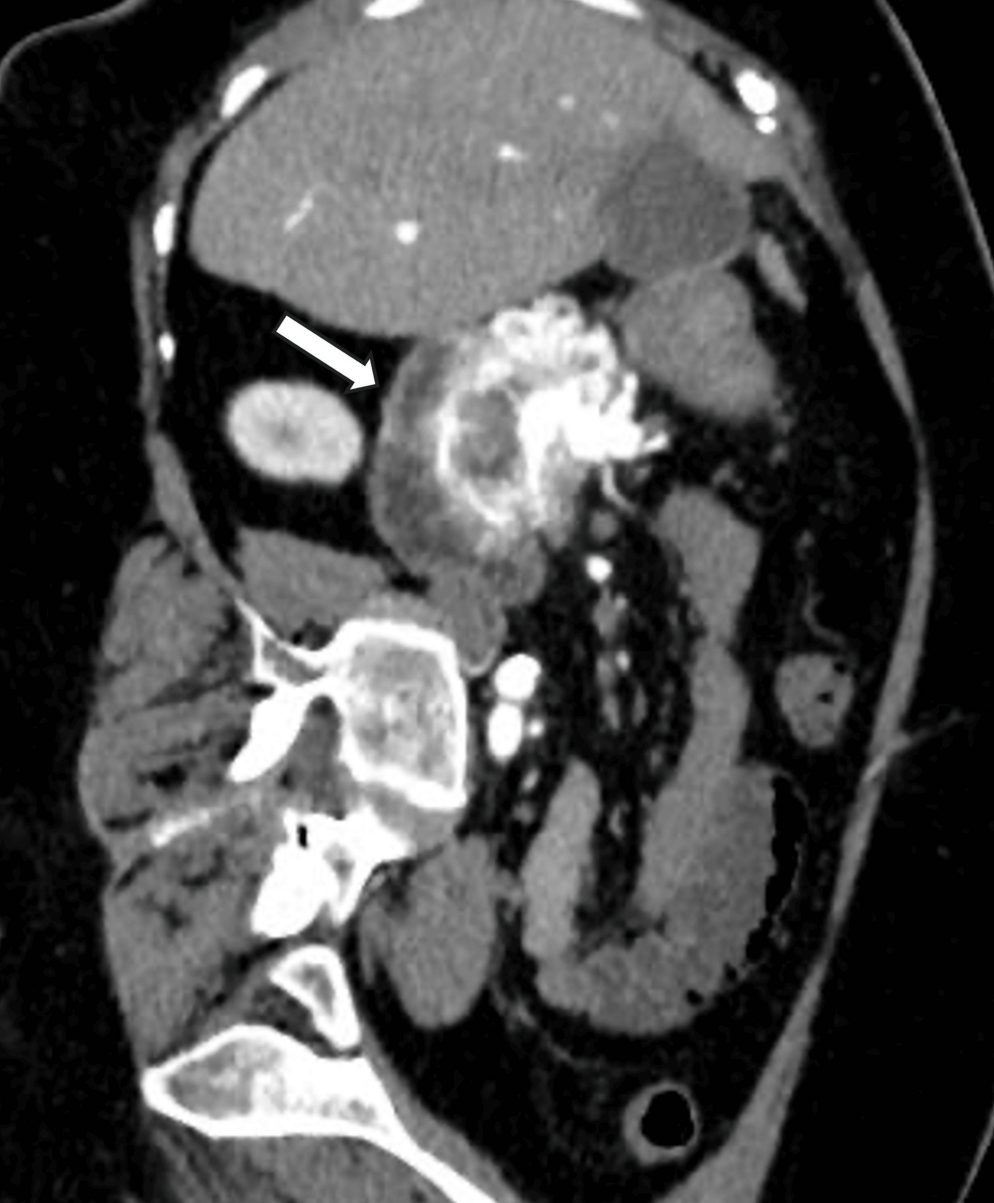
CT showing pancreatic head mass without duodenal invasion Contrast-enhanced CT of the abdomen, oblique reconstruction, shows a solid mass in the pancreatic head that contacts and displaces the first and second portions of the duodenum, without associated mural thickening, luminal narrowing, or obstruction to suggest direct invasion (arrow). CT: computed tomography

The lesion demonstrated peripheral arterial-phase enhancement with a hypodense central area compatible with necrosis and exerted a mass effect on the first and second portions of the duodenum without luminal obstruction or mural invasion (Figure 2).

**Figure 3:**
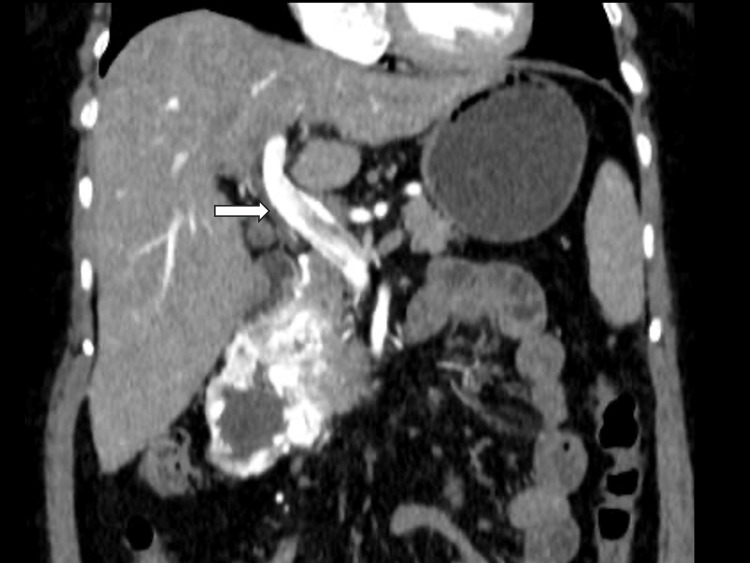
Early portal vein opacification Contrast-enhanced CT scan of the abdomen, coronal reconstruction in the arterial phase, demonstrating a markedly hyperdense portal vein (arrow) due to early contrast opacification, a finding that reflects increased flow and tumor-associated vascularity. CT: computed tomography

Prominent gastroduodenal veins and a hyperdense appearance of the portal vein in the arterial phase were noted, consistent with tumor hypervascularity. No regional lymphadenopathy or signs of peritoneal spread were identified (Figure 3).

Incidental findings included a mildly lobulated hepatic contour suggestive of early chronic liver disease, as well as a small amount of free intraperitoneal fluid. A non-contrast chest CT showed no pulmonary nodules, pleural effusion, or lymphadenopathy. Given the hypervascular pattern and location in the pancreatic head, the initial radiologic impression was that of a pancreatic NET.

**Figure 4:**
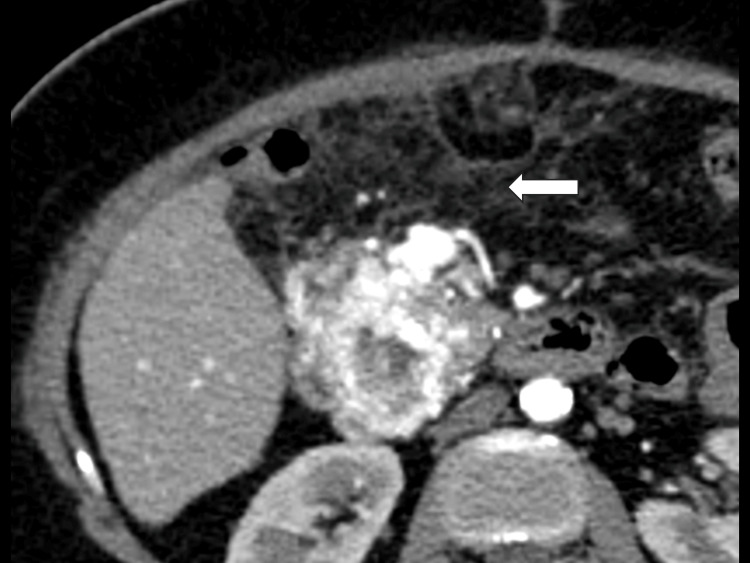
Follow-up imaging showing inflammatory changes Contrast-enhanced CT scan of the abdomen, axial plane in the early postoperative period after exploratory laparotomy and incisional biopsy of the pancreatic mass, again demonstrating a heterogeneously hyperenhancing lesion in the pancreatic head without significant change in size, with increased attenuation and stranding of the surrounding peripancreatic and mesenteric fat (arrow), findings compatible with inflammatory and postsurgical changes. CT: computed tomography

Three months after the initial presentation, a laparotomy with intent to resect the mass was performed. However, shortly after resection of a tissue sample from the lesion, the procedure was complicated by significant hemorrhage related to the marked hypervascularity of the tumor, making safe continuation of the resection unfeasible. The surgery was therefore aborted after achieving hemostasis, and an incisional biopsy of the mass was obtained. In the early postoperative period, follow-up contrast-enhanced CT demonstrated persistence of a hypervascular mass with similar characteristics, accompanied by postoperative inflammatory changes in the adjacent tissues and no evidence of nodal or metastatic disease (Figure 4).

**Figure 5:**
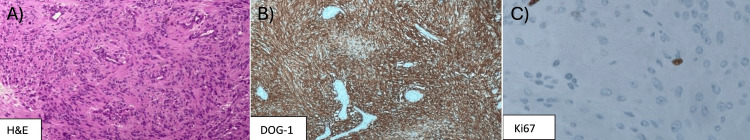
Histopathological and immunohistochemical study of the pancreatic head tumor (A) Hematoxylin and eosin–stained section showing a mesenchymal neoplasm with a mixed pattern of spindle and epithelioid cells arranged in intersecting fascicles, without evident necrosis. (B) DOG1 immunostaining demonstrating strong, diffuse membranous and cytoplasmic positivity in the neoplastic cells, consistent with a gastrointestinal stromal tumor. (C) Ki-67 immunostaining showing a low proliferative index of approximately 1%, supporting a low-risk GIST. H&E: hematoxylin and eosin; GIST: gastrointestinal stromal tumor

Histopathologic examination of the pancreatic incisional biopsy revealed a mesenchymal neoplasm with a mixed spindle-cell and epithelioid pattern, eosinophilic cytoplasm, and elongated nuclei, with a mitotic count of one per 5 mm², corresponding to a very low mitotic activity well below five mitoses per 50 high-power fields, and no tumor necrosis in the sampled tissue. The biopsy margins were involved by neoplastic cells. The immunohistochemical panel showed strong positivity for DOG1 and negativity for cytokeratin AE1/AE3, desmin, CD31, CD56, and S100. The Ki-67 proliferative index was close to 1%. These findings were interpreted as compatible with a GIST of mixed spindle-cell and epithelioid morphology and low histologic risk (G1) in the sampled tissue (Figure 5).

The liver biopsy showed macrovesicular steatosis and ballooning degeneration with periportal fibrosis stage F2 and a Nonalcoholic Fatty Liver Disease Activity Score of 6, consistent with steatohepatitis with activity. Cytologic examination of the ascitic fluid, evaluated with Papanicolaou stain and cell block preparation, was negative for malignancy and revealed reactive mesothelial cells in a chronic inflammatory background.

The case was discussed at a multidisciplinary conference, where a second intervention with curative intent was agreed upon, aiming for complete tumor resection with microscopically negative margins while preserving vascular structures and duodenal patency as far as possible. A pancreaticoduodenectomy (Whipple procedure) and treatment with imatinib were proposed. However, the patient declined both Whipple surgery and tyrosine kinase inhibitor therapy and opted for conservative management with close clinical follow-up. At the time of this report, she reports a noticeable decrease in abdominal distension and abdominal pain.

## Discussion

GISTs that primarily involve the pancreas represent an exceptional entity within the spectrum of EGISTs. Early descriptions noted that only a few cases of pancreatic EGIST had been reported in the literature, almost always located in the head of the pancreas and behaving as high-risk tumors [8,9]. Subsequently, larger series and reviews have confirmed the rarity of this location [5,10-12]. Multiple series have shown that abdominal pain is the most common symptom and that the head and body of the pancreas account for most tumors [5]. In this context, the case we present, a 63-year-old woman with a roughly 6-cm mass in the pancreatic head and a low mitotic index, fits the pattern of location and size previously described but exhibits low-risk histologic features that are uncommon in this topography [5,8].

From an imaging perspective, most pancreatic EGISTs present as solid or solid-cystic masses with intense enhancement after contrast administration and, in some cases, central areas of necrosis or cystic degeneration [9-11,13-15]. In several reports, these lesions were initially interpreted as NETs or other hypervascular solid tumors because of their arterial enhancement pattern [10,12,15]. Beltrame et al. described a case of pancreatic EGIST in which CT suggested a non-functioning NET before resection [10], whereas Soufi et al. reported a pancreatic EGIST associated with pancreas divisum treated with a Whipple procedure, emphasizing the need to include this entity in the differential diagnosis of solid pancreatic masses [12]. More recent studies focusing on the imaging characteristics of pancreatic EGISTs describe complex enhancement patterns, including peripheral enhancement and prominent intratumoral vessels, which may help suggest the diagnosis [15]. By contrast, previous CT series have shown that most pancreatic NETs present as small, well-circumscribed intraparenchymal nodules with intense arterial-phase enhancement and only subtle or even inapparent changes on later phases; atypical appearances, such as exophytic growth, predominantly cystic morphology, or extensive central necrosis, have been reported but are distinctly less frequent [16]. Pancreatic duct dilatation has traditionally been considered unusual in this setting and mainly related to large masses compressing the main duct; however, more recent data indicate that serotonin-producing NETs can also cause fibrotic strictures with upstream ductal dilatation and distal atrophy [17]. By comparison, our patient had a bulky pancreatic head mass with lobulated contours, marked peripheral enhancement and central necrosis, and no biliary or pancreatic duct dilatation, a combination that, together with the absence of lymphadenopathy, aligns more closely with published descriptions of pancreatic EGIST and helps to explain the preoperative diagnostic difficulty.

On histopathologic evaluation, pancreatic EGISTs usually display spindle-cell or mixed morphology and express classic markers such as CD117 and CD34, with variable rates of positivity for smooth muscle actin (SMA) and S100 [5,8,9,11,13,14]. The incorporation of DOG1 as an immunohistochemical marker has markedly improved diagnostic sensitivity, particularly in tumors with weak or negative KIT expression [7,15]. A series of EGISTs reports very high rates of DOG1 positivity and recommends its use in combination with CD117 to increase diagnostic certainty [5,13-15]. In our case, strong DOG1 positivity, together with mixed spindle-cell and epithelioid morphology and a low mitotic index, allowed us to establish the diagnosis of low-risk pancreatic EGIST and to integrate the imaging findings within a coherent diagnostic framework. Taken together, these data support the inclusion of EGIST in the differential diagnosis of hypervascular masses of the pancreatic head and underscore the value of correlating imaging, histology, and immunohistochemistry.

Although the mitotic index and the absence of necrosis in the biopsy supported a low-risk category, the tumor measured nearly 6 cm and arose in an extragastric site, factors that have been associated with a higher propensity for recurrence and lower disease-free survival compared with gastric GISTs of similar size and mitotic activity [1,5]. In addition, most GISTs harbor activating mutations in KIT or PDGFRA, leading to constitutive tyrosine kinase signaling and providing a clear biological rationale for the use of KIT-targeted agents such as imatinib in unresectable, metastatic, or high-risk disease [1,3]. Molecular testing for KIT and PDGFRA mutations was not performed in this patient, which represents a limitation; however, the classic morphology and immunophenotype strongly supported the diagnosis of pancreatic GIST and informed our therapeutic considerations. In our case, the combination of low mitotic index, large tumor size, pancreatic location, and incomplete surgical management prompted multidisciplinary discussion of imatinib as a potential systemic therapy option should progression occur, even though the patient ultimately declined tyrosine kinase inhibitor treatment.

## Conclusions

Primary GIST of the pancreatic head is an extremely rare entity among solid pancreatic tumors. It may present as a hypervascular mass with central necrosis and duodenal compression, favoring an initial suspicion of a NET or other hypervascular neoplasms. In the case presented, correlation of the CT findings with the mixed mesenchymal morphology and the immunohistochemical profile, characterized by strong DOG1 positivity and negativity for epithelial, muscular, and neural markers, enabled us to establish the diagnosis of low-risk GIST. This integrated approach is essential to guide therapeutic decision-making and oncologic follow-up and underscores the importance of considering pancreatic EGISTs in the differential diagnosis of hypervascular solid masses of the pancreatic head.

Beyond its rarity, this case underlines several practical considerations for daily practice. The marked hypervascularity of the tumor and the severe bleeding encountered at laparotomy illustrate how pancreatic EGISTs can pose substantial intraoperative hemostatic challenges, reinforcing the need for careful preoperative planning by surgical and anesthetic teams. In addition, a large pancreatic head mass with persistent vivid enhancement, central necrosis, and absence of biliary or pancreatic duct dilatation should prompt clinicians to contemplate pancreatic EGIST as an alternative to a NET when interpreting cross-sectional imaging. Finally, the decisive contribution of DOG1 immunostaining in this case emphasizes the central role of a targeted immunohistochemical panel in resolving NET-like imaging dilemmas and securing an accurate stromal tumor diagnosis.
